# Adrenal insufficiency as a cause of hypertransaminasemia and hyperferritinemia: case report and review of the literature

**DOI:** 10.1097/j.pbj.0000000000000269

**Published:** 2024-10-25

**Authors:** Bruna Silva, Catarina A. Pereira, Catarina Cidade-Rodrigues, Catarina Chaves, Alexandra Araújo, Ana Saavedra, Cláudia Machado, Mariana Martinho, Vânia Gomes, Margarida Almeida, Filipe M. Cunha

**Affiliations:** a Endocrinology Department, Centro Hospitalar do Tâmega e Sousa, Penafiel, Portugal; b Endocrinology Department, Centro Hospitalar, Vila Nova de Gaia/Espinho, Portugal

## Introduction

Adrenal insufficiency (AI) is a rare and potentially life-threatening condition. The diagnosis is not always straightforward. Atypical presentations of AI may include elevated transaminase levels or, rarely, high ferritin and liver iron deposits. There are only around 21 cases reported in the literature of hypertransaminasemia as the main finding in patients with AI, and there is just one showing iron deposits in a liver biopsy.^1-7^ The exact reasons for these findings remain uncertain. We report a patient who presented with high levels of transaminases and ferritin as the first manifestation of AI to highlight this rare, but important, association.

## Case report

**Figure 1.**
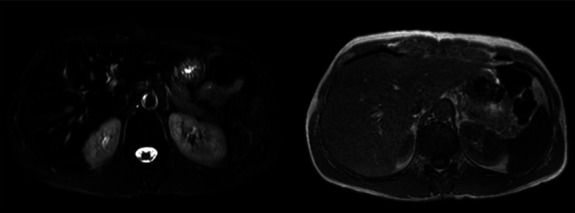
Abdominal magnetic resonance scan (left side: T2 sequence with fat suppression; right side: T1 sequence) showing signs of hepatic iron overload.

We report a 34-year-old man presented with consistently high liver transaminase levels. He had been experiencing anorexia, fatigue, weight loss, epigastric pain, nausea, and vomiting since the past year. His history included the occasional Raynaud phenomenon, and he had no history of alcohol consumption or medication use. His body mass index (BMI) was 21.6 kg/m^2^, with a blood pressure of 90/50 mmHg and a heart rate of 80 beats/minute. The laboratory measurements are presented in Table 1. His hemoglobin, creatinine, and potassium levels were elevated. His aspartate aminotransferase (AST) and alanine aminotransferase (ALT) values reached 88 U/l and 106 U/l, respectively, without alteration of other liver function tests. Ferritin levels were increased, peaking at 1555 ng/ml (normal range <454 ng/ml), without other iron parameter abnormalities. Common causes of liver abnormalities such as B and C hepatitis, autoimmune liver diseases, Wilson disease, and alpha-1 antitrypsin deficiency were ruled out. Thyroid function was normal, and thyroid peroxidase antibodies were negative. There were no abdominal ultrasound abnormalities while the computed tomography scan revealed hepatomegaly. Further investigation with abdominal magnetic resonance imaging showed a slight iron deposition in the reticuloendothelial system without significant steatosis (Fig. 1). Genetic testing for hemochromatosis was negative. In January 2022, he was hospitalized because of acute kidney failure and type 4 tubular acidosis. He was prescribed sodium bicarbonate and cation exchange and discharged to proceed the investigation. In May 2022, he presented to the emergency department with vomiting, nausea, hypotension, and symptomatic hypoglycemia (45 mg/dL). Skin hyperpigmentation was observed, along with elevated creatinine levels of 1.6 mg/dl, hyponatremia of 133 mmol/L, and hyperkalemia of 5.9 mmol/L (light gray column in Table 1). The diagnosis of primary adrenal insufficiency was confirmed with very low morning serum cortisol levels (<0.4 mcg/dL) and elevated morning serum adrenocoticotropic hormone (ACTH) levels of 674 pg/mL. In addition, aldosterone levels were <7 pg/dL, and the direct renin concentration was 134.4 pg/ml. Adrenal antibodies were positive. Treatment with hydrocortisone was initiated, resulting in rapid improvement of symptoms and laboratory findings (dark gray column in Table 1), including ferritin and transaminase levels and creatinine and electrolytic abnormalities.

**Table 1:** Laboratory findings on our patient since the beginning of the first symptoms.

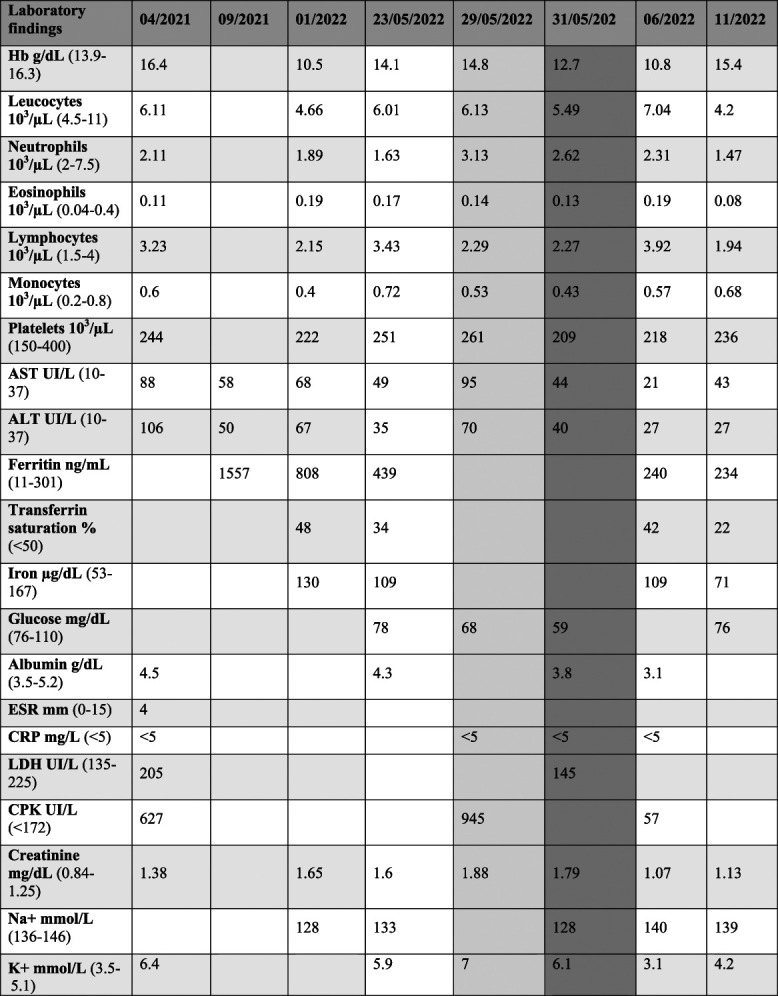

The light gray column represents blood analysis at the admission, and the dark gray one represents the day after the first hydrocortisone administration.

AST, aspartate aminotransferase; ALT, alanine aminotransferase; CPK, creatine phosphokinase; CSR, C-reactive protein; ESR, erythrocyte sedimentation rate; Hb, hemoglobin; Leuc, leucocytes; LDH, lactate dehydrogenase; Na+, sodium; Neu, neutrophiles; K+, potassium.

## Discussion

We report a case of primary adrenal insufficiency (PAI) presenting with hypertransaminasemia and hyperferritinemia. As far as we know, there are only 21 cases reported of AI: 20 were related to PAI and 1 was associated with the chronical use of corticosteroids.^1-3,6-10^ Only 1 presented with increased ferritin levels and hepatic hemosiderosis.^11^ In the reported cases, 27% of patients experienced an adrenal crisis at the time of diagnosis. Anti-adrenal antibodies were positive in 88% of the tested cases. 5 patients underwent liver biopsy: 2 showed periportal lymphocytic infiltration, 1 had enlarged portal tracts and hydropic degeneration of hepatocytes, another had minimal inflammation of the liver, and no abnormalities were found in the other.^2,5^ The mean AST and ALT levels were 166 U/L and 192 U/L, respectively. In 90% of the cases, this elevation was mild to moderate, only 1 case showing severe elevation and acute hepatic failure.^6^ The mean time from symptom onset or transaminase elevation to diagnosis was 440 days, and transaminase levels returned to normal within 23 days of starting corticosteroid treatment. The mechanism behind adrenal insufficiency-induced transaminase elevation remains unclear. While autoimmune disorders such as hepatitis, celiac disease, or thyroid issues associated with AI can cause liver damage,^12,13^ this does not explain transaminase elevation in corticosteroid-related cases. Nevertheless, in most of the patients reviewed, autoimmune hepatitis-related antibodies were negative and no other autoimmune disorders were found. This was the case of our patient as well, which makes this hypothesis less plausible. Another possible mechanism postulated was hepatocyte hypoxia caused by chronic hypoperfusion or weight changes.^14^ However, hypotension is not usually severe enough to cause ischemic injury, except in cases of severe hypotension during an adrenal crisis. A third hypothesis attributes to the effects of general cortisol deficiency.^15^ A decrease in cortisol can lead to inflammation, with leukocyte recruitment, cytokine release, lymphocytic infiltration in hepatocytes, and consequent immune-mediated liver damage.^7^ Furthermore, an immune-mediated liver damage is supported by infiltration of the portal zones reported in previous reports.^11^ In addition, the case involving the discontinuation of corticosteroid therapy supports this hypothesis. In fact, this could also account for the higher ferritin levels observed in patients with adrenal insufficiency.^16^ Cortisol suppresses inflammation, and its deficiency might result in chronic unmodulated inflammatory response to various stimuli,^17^ leading to increased ferritin levels, an acute phase reactant.^18^ Retrospectively, our patient had symptoms and other laboratory abnormalities that suggested adrenal insufficiency as their cause. Anorexia, weight loss, and gastrointestinal symptoms are common in adrenal insufficiency and in liver disease. The type 4 renal tubular acidosis can be caused by mineralocorticoid deficiency that occurs in PAI. While, individually, these findings provided little diagnostic assistance, collectively, they strongly suggested PAI.

## Conclusion

The reported cases provide evidence that elevated transaminases and ferritin levels can be observed in patients with adrenal insufficiency. This condition needs to be considered in face of unexplained persistent elevation of transaminases, accompanied by hyperferritinemia or not. Early diagnosis can prevent adrenal crisis and unnecessary liver biopsies.
